# Effect of wetland management: are lentic wetlands refuges of plant-species diversity in the Andean–Orinoco Piedmont of Colombia?

**DOI:** 10.7717/peerj.2267

**Published:** 2016-08-16

**Authors:** Johanna I. Murillo-Pacheco, Matthias Rös, Federico Escobar, Francisco Castro-Lima, José R. Verdú, Germán M. López-Iborra

**Affiliations:** 1 I.U.I. CIBIO—Universidad de Alicante, Alicante, Spain; 2 Facultad Ciencias Básicas e Ingeniería, Universidad de los Llanos, Grupo de investigación BIORINOQUIA, Villavicencio, Colombia; 3 Instituto Politécnico Nacional, CONACYT Research Fellow, CIIDIR, Oaxaca, Mexico; 4 Red de Ecoetología, Instituto de Ecología AC, Xalapa, Mexico; 5 Villavicencio, Meta, Colombia; 6 Departamento de Ecología, Instituto de Investigación IMEM Ramón Margalef, Universidad de Alicante, Alicante, Spain

**Keywords:** Archipelago reserve, Compositional similarity, Hill numbers, Meta Piedmont forest, Wetland type and origin, Woody and aquatic plants

## Abstract

Accelerated degradation of the wetlands and fragmentation of surrounding vegetation in the Andean–Orinoco Piedmont are the main threats to diversity and ecological integrity of these ecosystems; however, information on this topic is of limited availability. In this region, we evaluated the value of 37 lentic wetlands as reservoirs of woody and aquatic plants and analyzed diversity and changes in species composition within and among groups defined according to management given by: (1) type (swamps, heronries, rice fields, semi-natural lakes, constructed lakes and fish farms) and (2) origins (natural, mixed and artificial). A total of 506 plant species were recorded: 80% woody and 20% aquatic. Of these, 411 species (81%) were considered species typical of the area (Meta Piedmont distribution). Diversity patterns seem to be driven by high landscape heterogeneity and wetland management. The fish farms presented the highest diversity of woody plants, while swamps ranked highest for aquatic plant diversity. Regarding wetland origin, the artificial systems were the most diverse, but natural wetlands presented the highest diversity of typical species and can therefore be considered representative ecosystems at the regional scale. Our results suggest that lentic wetlands act as refuges for native vegetation of Meta Piedmont forest, hosting 55% of the woody of Piedmont species and 29% of the aquatic species of Orinoco basin. The wetlands showed a high species turnover and the results indicated that small wetlands (mean ± SD: size = 11 ± 18.7 ha), with a small area of surrounding forest (10 ± 8.6 ha) supported high local and regional plant diversity. To ensure long-term conservation of lentic wetlands, it is necessary to develop management and conservation strategies that take both natural and created wetlands into account.

## Introduction

The vegetation associated with wetlands is one of the most distinctive characteristics of aquatic ecosystems (Burton, Tiner & Gene, 2009; Mitsch & Gosselink, 2015). Plants are an important component of the dynamic of the aquatic systems and are considered an efficient indicator of ecological integrity (Cronk & Fennessy, 2001; Clément & Proctor, 2009). However, it is known that biodiversity in wetlands is related to land use in the surrounding landscape in which they are immersed, as well as the type and intensity of management practiced (Wang et al., 2008; Mitsch & Gosselink, 2015). Wetlands are known as strategic ecosystems for biodiversity (MEA, 2005; Zedler & Kercher, 2005), and they contribute to the maintenance of ecological functions across natural and human-dominated landscapes (e.g., in rice fields Rolon et al., 2008; Rolon & Maltchik, 2009). Nevertheless, the role of wetlands as biodiversity reservoirs remains relatively poorly understood in Neotropical regions, particularly in the wetlands of the Orinoco river basin region (e.g., Díaz & Rosales, 2006; Fernández, Bedoya & Madriñán, 2015).

The Andean Piedmont of the Colombian Orinoco river basin once formed a continuous corridor of vegetation between the Eastern cordillera of the Andes, and the savannah ecosystems of the Llanos Orientales (Bates, 1948; Molano, 1998). This was a typical rainforest with swamplands that formed and maintained multiple wetlands (Bates, 1948) within the region known as “Villavicencio pleistocene refuge” (Hernández et al., 1992). This region harbors over 3,000 plant species (Minorta-Cely & Rangel-Ch, 2014) and is therefore considered one of the most diverse areas in northern South America. The Andean–Orinoco Piedmont has been heavily fragmented (Etter, McAlpine & Possingham, 2008), and significant portions of the remaining vegetation are concentrated around wetlands. In this region, the natural wetlands (i.e., swamps and diverse flooded lands) and their surrounding vegetation have been reduced as a result of the expansion of the agricultural frontier, the urbanization and changes of land cover (i.e., Romero-Ruiz et al., 2004; Romero-Ruiz et al., 2009). Currently, lentic wetlands are distributed as isolated patches within an urban and rural landscape matrix, forming islands of aquatic ecosystems surrounded by Meta Piedmont vegetation, partly connected by riparian and flooded forests.

Wetlands are a conservation priority at regional (Lasso et al., 2014), national (MMA, 2002) and global level (MEA, 2005; Mitsch, Bernal & Hernandez, 2015), and diversity studies play a key role in order to take efficient actions for their conservation and management (Ramsar, 2007; Mitsch & Gosselink, 2015) Wetland management and the different human activities involved modify plant diversity and composition (McKinney & Lockwood, 1999). One effect of the fragmentation and simplification of forests is the dilution and biotic homogenization of native plant species assemblages (e.g., Magee et al., 1999; Chen et al., 2010). On the other hand, biotic differentiation is also possible as a consequence of isolation of the vegetation remnants (Arroyo-Rodríguez et al., 2013), and management practices (i.e., creation of wetlands, living fences, gardens), bringing with it the introduction of native and foreign species (Gallardo et al., 2015) which may affect negatively the diversity of typical species and the functionality of wetlands (Hager, 2004; Ervin et al., 2006; Lambdon, Lloret & Hulme, 2008).

The importance of type and origin of wetlands as drivers of biotic homogenization and differentiation of plant diversity is poorly known. In highly modified landscapes as in Andean Piedmont, vegetation is reduced to small fragments, many of them associated with lentic wetlands. Several studies highlight the role that these remnants have for the maintenance of plant diversity regardless whether these are natural (Flinn, Lechowicz & Waterway, 2008) or created wetlands (Chester & Robson, 2013). They may be considered as a refuge to species, in such a way that their associated vegetation could be acting as small forest patches that contribute highly to the regional diversity (Arroyo-Rodríguez et al., 2009).

In this study we evaluate the role of natural and created lentic wetlands as a refuge of plant diversity of the Meta Piedmont biome, and the influence of the wetland management in the diversity of woody and aquatic plants, focusing on two questions: (1) are lentic wetlands, despite its size, refuges of plant diversity; and, (2) how do the diversity and composition of plants associated with lentic wetlands change according to their management given by type and origin? We expect that wetland type has a strong influence on local and regional plant diversity, that natural wetlands retain the greatest plant diversity of Andean Piedmont and that lentic wetlands are potential reservoirs of plant diversity. This study presents the first estimates of plant diversity in lentic wetlands in the region, and aims to be a scientific baseline for the sustainable management and conservation of these wetlands and their associated vegetation.

## Methods

### Study area

**Figure 1:**
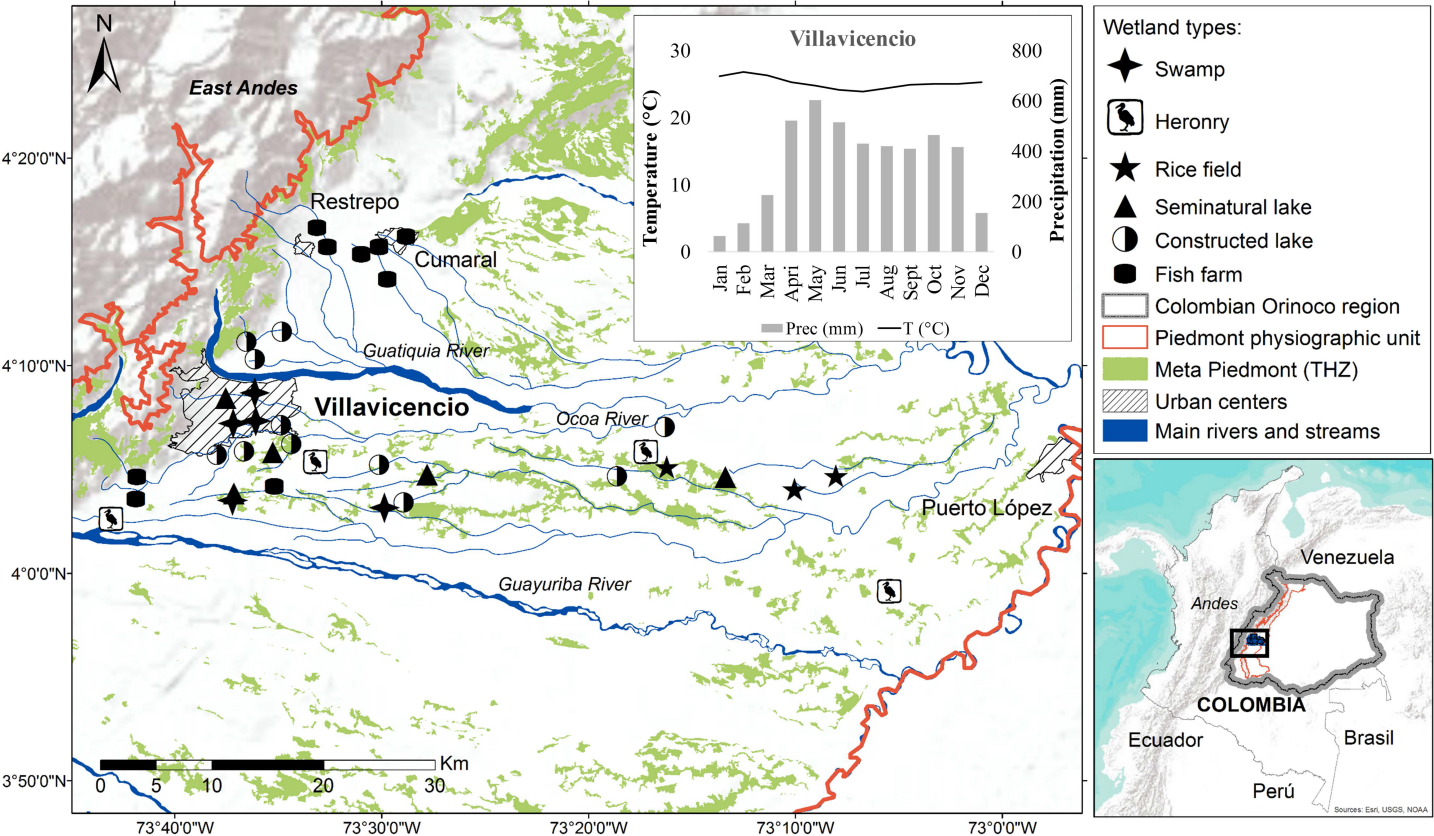
Localization of lentic wetlands at the Meta Piedmont in Colombian Andean–Orinoco region. Municipalities of Villavicencio, Restrepo, Cumaral and Puerto López with urban center. The climograph shows the mean temperature (line) and precipitation (bars) of Villavicencio municipality.

Andean Piedmont is a subregion of the Colombian Orinoco river basin, located in the East Andes in a transitional confluence area between the Andean mountains, and the Amazon and Savannah ecosystems, located within the physiographic unit known as Piedmont (Molano, 1998; Romero-Ruiz et al., 2004; Rosales, Suárez & Lasso, 2010; Minorta-Cely & Rangel-Ch, 2014). Our study area is found within the biome known as Meta Piedmont tropical humid zonobiome (THZ) (Romero-Ruiz et al., 2004), this covers 0.3% (83.493 ha) of the natural ecosystems of the Orinoco river Basin and is considered one of the most diverse ecosystems per unit area in the region. It is characterized by an altitude range of between 200–1,100 m a.s.l., tropical very humid warm climate, mean annual temperature of 25 ° C and mean annual rainfall ranging from 2,700 to 4,600 mm, with the highest precipitation between April and June with monthly means superior to 500 mm (Minorta & Rangel, 2014) (see climograph in Fig. 1).

This study was performed in the municipalities of Villavicencio, Puerto López, Restrepo and Cumaral, Department of Meta (4°3′14.7^′′^N–73°42′56.8^′′^W and 3°59′41.1^′′^N–73°5′12.2^′′^W) (Fig. 1). Sampling was carried out in 37 lentic wetlands. They were grouped according to the type and origin of the wetlands, following our own proposed classification based on MMA (2002), Ramsar (2012) and Lasso et al. (2014).

Wetland management was studied in six types of wetlands: (1) swamps (SW) are natural forest wetlands with emergent woody vegetation, they are flooded lands without a defined open body of water, associated with sources of other wetlands like streams or rivers; (2) heronries (HC) are natural swamplands with flooded areas and are characterized by the congregation of mixed species of breeding aquatic birds, specially herons and egrets; (3) rice fields (RF) are farms of irrigated rice crops that are mixed with natural water sources, channels and sometimes small dams, also they have fencerows with vegetation; (4) semi-natural lakes (SNL) were created after some management on natural wetlands that retain features of them and have tourist and recreational purposes; (5) constructed lakes (CL) are artificial wetlands that were built in its entirety and are used as recreational lakes and dams; (6) fish farms (FF) are generally rectangular shaped ponds, created for aquaculture of freshwater fish production (see Table 1, for characteristic of wetlands).

**Table 1:** Lentic wetlands studied. Size refers to total area of water surface (total 424 ha) and forest coverage (total 435 ha) in a 500 m buffer calculated from centroid of each wetland. Mean ± SD shown in brackets.

Wetland type	Origin	No. of wetlands	Size (ha)	Forest (ha)
Swamps (SW)	Natural	6	76.7 (12.8 ± 17.8)	68.7 (11.4 ± 8.6)
Heronries (HC)	Natural	4	6.9 (1.7 ± 1.2)	19.1 (4.8 ± 6.8)
Rice fields (RF)	Mixed	3	188.3 (62.8 ± 18.9)	27.9 (9.3 ± 15.6)
Semi-natural lakes (SNL)	Mixed	6	25.5 (4.2 ± 2.6)	77.7 (12.9 ± 7.2)
Constructed lakes (CL)	Artificial	9	20.5 (2.3 ± 2.1)	95.4 (10.6 ± 7.4)
Fish farms (FF)	Artificial	9	106.2 (11.8 ± 15.9)	146.4 (16.3 ± 12.4)

We classified wetland types into three categories according to their origins: (1) natural: those that retain their original natural characteristics and where human intervention does not affect its natural dynamics (SW and HC); (2) mixed: those that have a natural origin but were transformed through human actions with extensive modification, so despite they can retain their natural features, they depend heavily on the constructed sections and facilities that regulate their hydric cycle (SNL and RF), and (3) artificial: wetlands that are completely human-made in places where no previous lentic wetland existed (CL and FF).

### Sampling

We studied plant formations of woody and aquatic species. The sampling was carried out through standardized methods in each wetland. Woody plants (i.e., trees and shrubs) were sampled in four linear 50 × 2 m transects (0.04 ha), located as close as possible to the wetland shore, within a 200 m buffer around, and with a minimum distance of 100 m between transects. All individuals with a diameter at breast height (DBH) ≥2.5 cm were recorded. Aquatic plants were sampled using ten 1 × 1 m plots placed in the flooded area of the wetland shore, where emergent vegetation was present (total of 320 plots, because in five wetlands we did not found aquatic species) with a minimum distance of 5 m between plots. In each plot, the percent cover of every species was measured. Only flowering plants (Angiosperms) and vascular cryptogams (Pteridophytes) were considered in this study. Species categorized as ‘typical’ were those having a distribution in the Meta Piedmont biome, and we refer to introduced exotic (from foreign countries) and native exotic species (with distribution in others native Colombian ecosystems) as ‘introduced’ plants. All plants were identified in the field by a specialist. The taxonomic species list is based on Cronquist (1981) and for introduced species on Alvarado & Gutiérrez (1997) and Cárdenas, Castaño & Cárdenas (2010).

### Data analysis

#### Inventory completeness

We used the sample coverage estimator proposed by Chao & Jost (2012) to estimate the accuracy of inventories in each wetland, and in each type and origin category of the wetlands, using the iNEXT package for R (Hsieh, Ma & Chao, 2016):

(1) \begin{eqnarray*}\hat {C}m= \left( 1- \frac{{f}_{1}}{n} \left[ \frac{(n-1){f}_{1}}{(n-1){f}_{1}+2{f}_{2}} \right] \right) \ast 100,\end{eqnarray*}

where *n* is the total number of plants, *f*_1_ and *f*_2_ are the numbers of singletons and doubletons of the sample. $\hat {C}m$ has values from 0 (minimal completeness) to 100% (maximum completeness), and indicates the proportion of the ecological community that is represented by the species recorded and is used as a measure of sampling completeness (Chao & Jost, 2012; Chao et al., 2014).

#### Species diversity

Diversity patterns were evaluated using Hill numbers ^*q*^*D* (details in Jost, 2006; Chao et al., 2014) for each wetland, within wetland type and origin, and among them. Hill numbers were calculated for order *q* = 0, corresponding to species richness (^0^*D*) which is not sensitive to abundances (Jost, 2006), and diversity of order *q* = 2, which is the inverse Simpson concentration index (^2^*D*), and can be interpreted as the number of ‘dominant’ species (Jost, 2010a)

For both orders (^0^*D* and ^2^*D*), diversity (*α*-diversity) was calculated in three ways: local alpha diversity in each wetland (*α*_*w*_), total alpha diversity corresponding to pooled diversity of each type and origin category (*α*), and mean alpha diversity ($\bar {\alpha }$) as average for each category of wetland type and origin. Finally, we obtained the regional (*γ*-diversity) as the total diversity of all wetlands.

Mean alpha diversity ($\bar {\alpha }$) among categories of type and origin of wetlands was compared based on its confidence intervals in which the absence of overlap between CI 95% indicated significant differences (Cumming, Fidler & Vaux, 2007). In order to compare the communities, we calculated the magnitude of the difference in diversity among communities, which is used to know how many times is one sample more diverse than another sample (Jost, 2006). The values of diversity in the two orders (*q* = 0 and 2) were compared directly and also using the percentage of species diversity difference magnitude (%MD) between types and origins of wetlands:

(2) \begin{eqnarray*}\text{%}\mathrm{MD}=[({\text{}}^{q}{D}_{\mathrm{ sample}}\gt S{-}^{q}{D}_{\mathrm{ sample}}\lt S)\ast 100{/}^{q}{D}_{\mathrm{ sample}}\lt S]\end{eqnarray*}

where ^*q*^*D*_sample_ > *S* is the sample with more diversity and ^*q*^*D*_sample_ < *S* is the sample of less diversity (modified from Cultid-Medina & Escobar, 2016).

To evaluate species inequality, we calculated the relative logarithmic inequality (RLI), which is the logarithmic transformation of the inequality factor *IF*_0,*q*_ (Jost, 2010b):

(3) \begin{eqnarray*}{RLI}_{0,q}= \frac{(In\hspace*{1.00006pt}I{F}_{0,q})}{(In\hspace*{1.00006pt}S)} ,\end{eqnarray*}

where *S* is the total alpha diversity and *IF*_0,2_ is the inequality factor (*IF*_0,2_=^0^*D*∕^2^*D*). When *RLI*_0,*q*_ is 0, it has maximum equality, when it is 1, it indicates maximum inequality (see details in Jost, 2010b).

#### Compositional similarity

We measured compositional similarity ^*q*^*CS* (Jost, 2007) within and among categories of type and origin wetlands

(4) \begin{eqnarray*}{\text{}}^{q}CS= \frac{ \left( {\text{}}^{q}{D}_{\alpha }/{\text{}}^{q}D\gamma - \frac{1}{N} \right) }{ \left( 1- \frac{1}{N} \right) } \end{eqnarray*}

where ^*q*^*Dα* is alpha diversity, ^*q*^*Dγ* is gamma diversity (corresponding to total alpha of each category), and *N* is the number of samples; ^*q*^*CS* = 0 when all samples are completely distinct, and ^*q*^*CS* = 1 when all are identical. When *q* = 0, and *N* = 2, it equals the Jaccard index, and for *q* = 2 the Morisita–Horn–Index (Jost, 2007).

## Results

We recorded a total of 506 vascular plant species from 85 families and 324 genera in the 37 lentic wetlands. 407 species were woody plants and 99 aquatic species. There were 411 (81%) typical species of Meta Piedmont and of the remaining 95 introduced species (19%), 43 corresponded to introduced exotic species and 52 to native exotic ones. Additionally, some of the species are categorized as threatened in red lists: *Cedrela odorata* as endangered in Colombia and Vulnerable (IUCN, 2013), and also the *Bactris gasipaes* and *Syagrus sancona* in the national category as Vulnerable (VU).

### Completeness and diversity patterns

The sampling coverage in each wetland was higher than 74%. Taking into account the wetlands according to the type and origin, completeness was greater than 92% (Table S1). Given the high inventory reliability, diversity comparisons were performed using the observed values.

**Figure 2:**
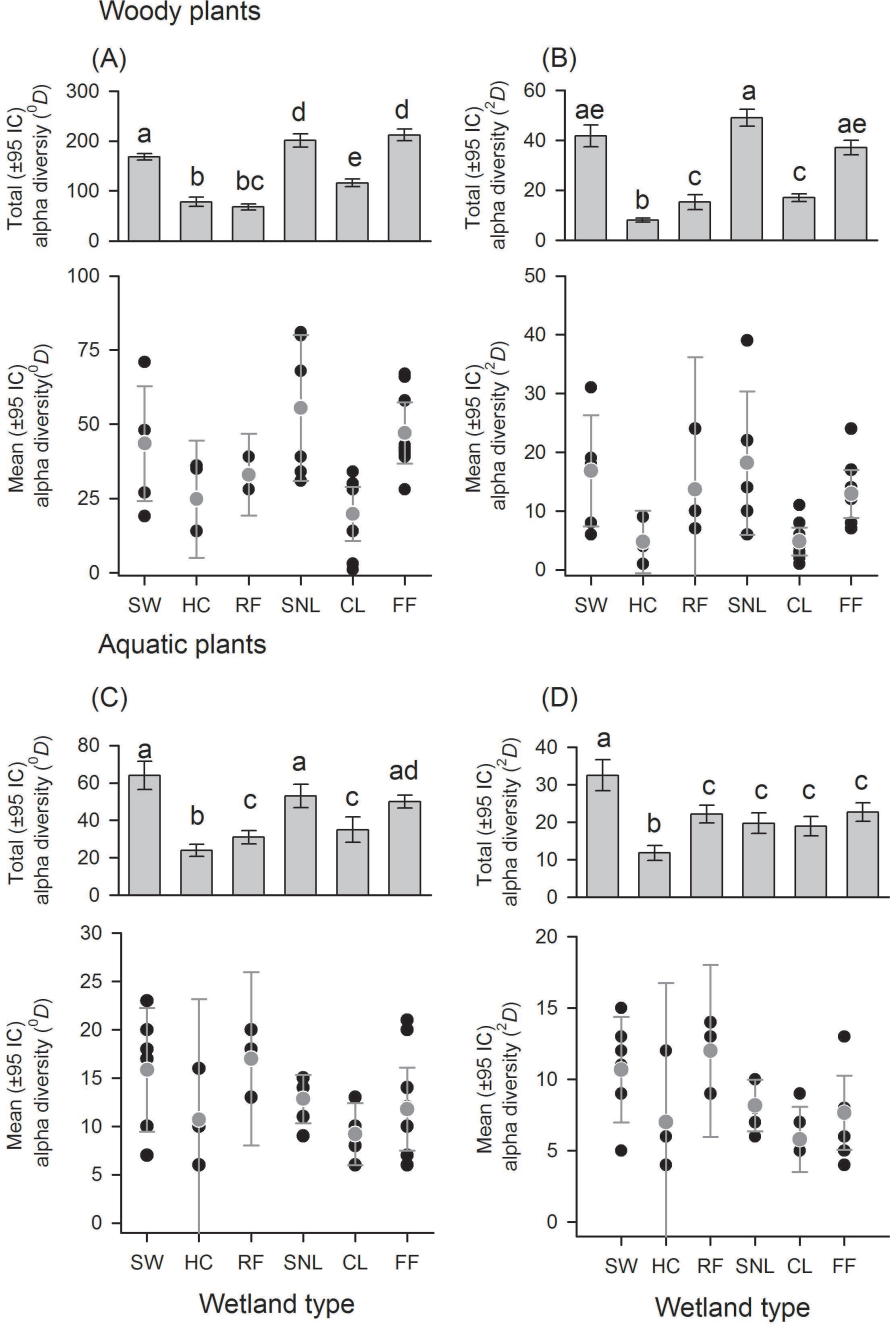
Alpha diversity of woody and aquatic plants according to wetland types. Wetland types: swamps (SW); heronries (HC); rice fields (RF); semi-natural lakes (SNL); constructed lakes (CL); fish farms (FF). Diversity using Hill numbers: species richness (^0^*D*) and dominant species (^2^*D*). Mean alpha diversity are gray points and local alpha diversity black points. Note that despite similar mean alpha diversity, the contribution of each wetland type to total alpha diversity is different.

**Figure 3:**
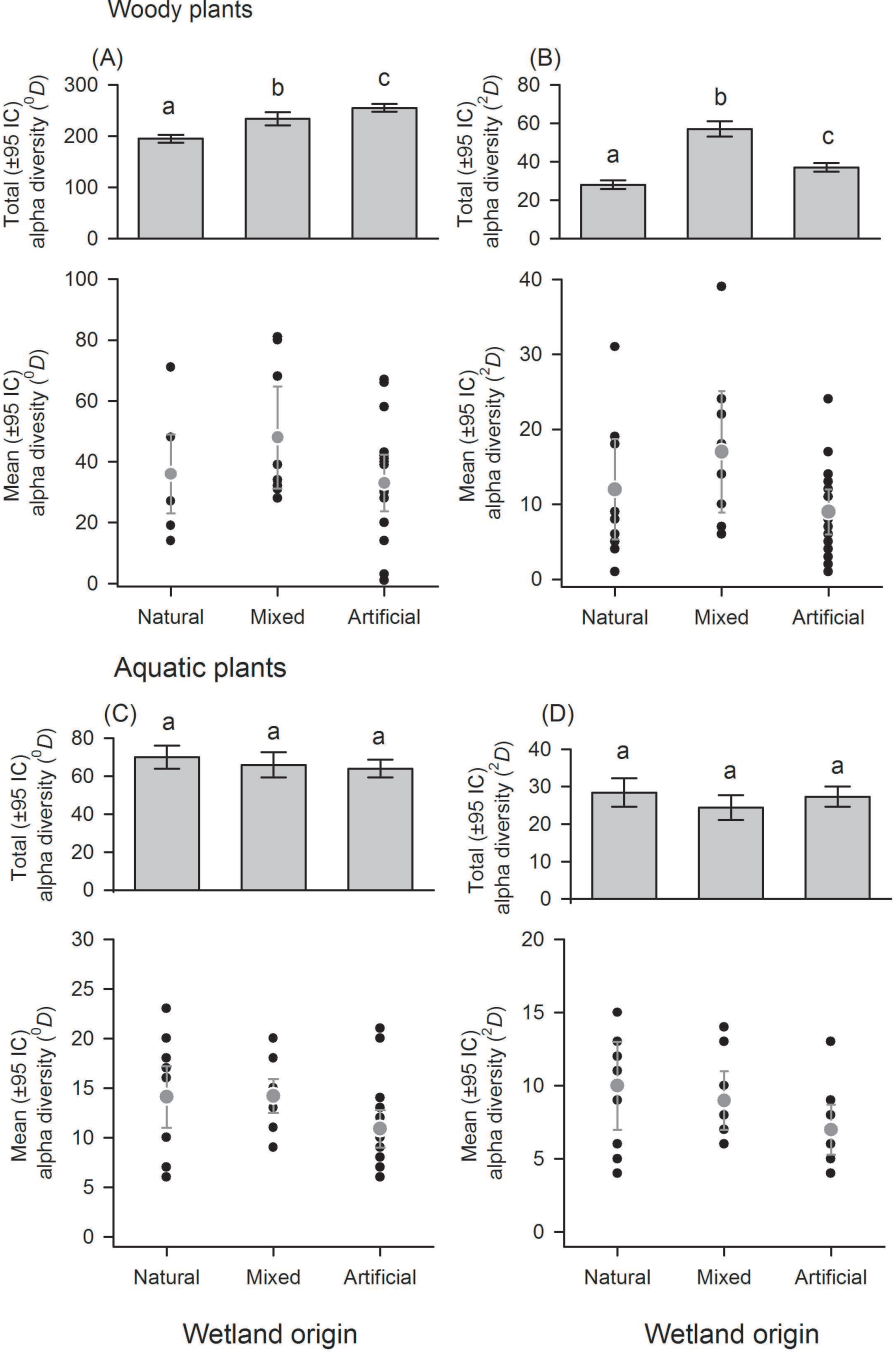
Alpha diversity of woody and aquatic plants within and among wetland origins. Diversity using Hill numbers: species richness (^0^*D*) and dominant species (^2^*D*). Mean alpha diversity are gray points and local alpha diversity black points.

There was a great variation in local diversity between wetlands in richness (^0^*D*) and dominant species (^2^*D*) (Figs. 2–3 and Tables S1–S2), being highest in the semi-natural lake SNL6 (Lake Bioparque Ocarros, 94 species) and lowest in the constructed lake CL6 (Lake La Tonga, 11 species, see Table S2). There were no statistical differences in the mean alpha diversity ($\bar {\alpha }$) of woody and aquatic plants for both wetland type and origin (Figs. 2–3 and Table S3). Total alpha diversity (*α*) showed differences among wetland types for both woody and aquatics plants (Figs. 2–3 and Table S2). Woody species richness (^0^*D*) was highest in FF (212 ± 12) and SNL (201 ± 13), and it was lowest in HC (78 ± 9) and RF (68 ± 6), presenting large richness difference magnitude between FF and RF (212%) as well as between SNL and RF (196%) (Fig. 2A). The pattern was similar regarding dominant species (^2^*D*), especially in HC, which showed the highest inequality (*RLI*_0,2_ = 0.52) (Fig. 2B and Table S1), being the most dominant species *Mabea trianae* and *Bactris corossilla* with 29% and 16% of all individuals.

In aquatic plants there were also marked differences in total alpha diversity in the two orders evaluated (^0^*D* and ^2^*D*), especially between SW and HC with a difference magnitude of 167% for species richness and 177% in terms of dominant species (Fig. 2C). In general, the inequality values were low. SNL was the wetland type with greater dominance (*RLI*_0,2_ = 0.25, Fig. 2D and Table S1), where the presence of *Ludwigia inclinata,* with 16% of their plant coverage, stands out.

With regard to wetland origin, woody plants in artificial wetlands had higher species richness (^0^*D*), mixed wetlands were more diverse regarding dominant species (^2^*D*), and natural wetlands were the least diverse (Figs. 3A and 3B), but had more inequality (*RLI*_0,2_ = 0.3). Species richness difference magnitude was 31% between artificial and natural wetlands, and dominant species difference magnitude was 101% between mixed and natural wetlands. In aquatic plants, the differences in diversity were low between origins (Figs. 3C and 3D), as well as the values of inequality (*RLI*_0,2_ < 0.24). However, natural wetlands had greater species richness (^0^*D*) and 14 species were exclusive in these.

### Compositional similarity (^*q*^*CS*)

**Figure 4:**
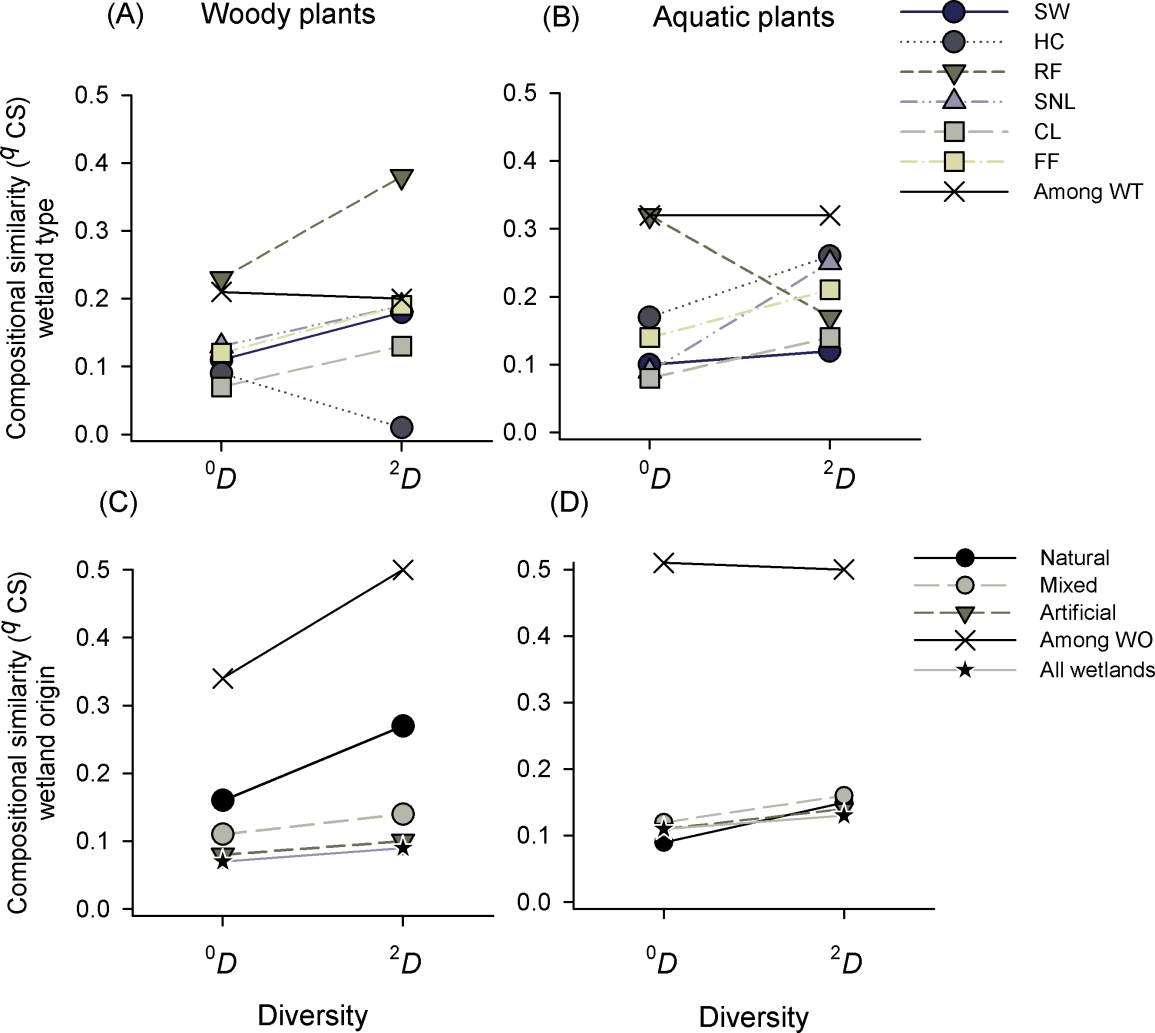
Compositional similarity (^*q*^*CS*) of species richness (^0^*D*) and dominant species (^2^*D*) of woody and aquatic plants within and among wetland types (WT) and origins (WO). Wetland types: Swamps (SW); heronries (HC); rice fields (RF); semi-natural lakes (SNL); constructed lakes (CL); fish farms (FF).

Compositional similarity in the whole landscape (i.e., for the entire set of wetlands studied) was low, but it showed a high variability within wetland types (Table S4). For both plant formations there was a high internal variability in the groups analyzed and ^*q*^*CS* was lower within groups than among groups (Figs. 4A and 4B). Taking into account the diversity of woody plants, constructed lakes (CL) were the wetland type with less similarity for all species (^0^*CS* = 0.07), and heronries (HC) for dominant species (^2^*CS* = 0.01), while rice fields (RF) showed higher similarity, which increased as *q* increased (^0^*CS* = 0.23 to ^2^*CS* = 0.38, Fig. 4A). The pattern was similar in aquatic plants, it showed low similarity values, especially in CL and with maximum values in RF (^0^*CS* = 0.08 and 0.32, respectively). All other wetland types had values lower than 0.17. Regarding dominant species, SW showed the lowest and HC the highest values (^2^*CS* = 0.1 and 0.26 respectively, Fig. 4B). Comparing CS of wetland types in woody and aquatic plants, we found a higher similarity among categories than within them in the two orders of similarity (^0,2^*CS*) (Figs. 4A, 4B and Table S4).

Compositional similarity regarding the origin of wetlands in woody plants was low within the categories (natural, mixed and artificial) and intermediate among them. Natural wetlands had the highest similarity, especially in dominant species ^0^*CS* = 0.16 and ^2^*CS* = 0.27) (Fig. 4C). In the same way, in aquatic plants the compositional similarity was low within each origin category (^*q*^*CS* < 0.16) and intermediate among them (^*q*^*CS* = 0.5) for both richness (^0^*CS*) and dominant species (^2^*CS*) (Fig. 4D and see Table S4).

## Discussion

This is the first comparative study on the diversity of plants in freshwater inland wetlands under different regimes of management in the Neotropical region. Although the total area of the 37 wetlands studied (424 ha) and their forest area (435 ha) associated represent less than 1% of wetland coverage in the Orinoco region and 1% of the Meta Piedmont tropical humid zonobiome (Romero-Ruiz et al., 2004), our results indicated that these wetlands make a considerable contribution to local and regional diversity of woody and aquatic plants. The diversity of plants found represents around 55% of the woody species of the Meta Piedmont forest (Carvajal et al., 2007), 12% of the flowering plants (Minorta-Cely & Rangel-Ch, 2014), and 29% of aquatic plants (Fernández, Bedoya & Madriñán, 2015) of the Colombian Orinoco river basin region. Typical Piedmont species showed high representation (81%), but the portion of introduced plants was considerable (19%), and had already an impact on diversity patterns and probably on the function of wetlands (Hager, 2004; Lambdon, Lloret & Hulme, 2008). The high diversity of the woody plants is related to forest of Meta Piedmont, where species of the Amazon and the Andes region converge, in addition to species associated with the eastern savannas (Molano, 1998; Fernández et al., 2010), while aquatic plant diversity is related to the high complexity and variety of aquatic ecosystems in the Orinoco region (Rial, 2014; Fernández, Bedoya & Madriñán, 2015). Therefore, removal of these remnants of the landscape could endanger the conservation of plants of a region characterized by its high biodiversity (Bates, 1948; Hernández et al., 1992; Molano, 1998; Fernández et al., 2010).

Contrary to our expectation, the origin of natural wetlands had low diversity both in richness and dominant species, which is associated to the fact that the heronries (HC) presented the lowest diversity between wetland types. This is probably due to the accumulation of guano caused by the congregation of birds, which may have led to a naturally caused impoverishment of diversity due to the high nitrogen content of excrements (Ayarzaguena, Pérez & Ramo, 1981), and it has been shown that a high bird activity can reduce the plant density of heronries (Weseloh & Brown, 1971). Additionally, they are strongly altered by livestock which modifies the structure of the surrounding vegetation, since the increase of cattle grazing can affect negatively the wetland diversity (Junk, 1993). On the contrary, swamps (SW) did not have such a low diversity, but lower than expected, as they are the most representative natural wetlands in the region (Lasso et al., 2014). The diversity of these wetlands can be related to the persistent human intervention along the time. Despite some swamps are locally protected, most of them are public lands, where chronic extraction of timber and urbanization processes in surrounding areas have been common (Machado, Rial & Lasso, 2011).

The finding that artificial wetlands have a greater diversity of woody plants can be explained by the fact that both fish farms (FF) and constructed lakes (CL) are created by private enterprises, where owners have particular interests. Most of these wetlands were built on relict forest. Meanwhile, mixed wetlands retain a high diversity of species typical of Piedmont because they were developed on natural wetlands for recreational (semi-natural lakes—SNL) and agricultural (rice fields—RF) purposes, and its management allows to keep a portion of the diversity of native plants. The use of agrochemicals is often associated with the decline of plant diversity (Rolon & Maltchik, 2009); although RF belonged to the group with higher diversity, we cannot exclude the possibility that without agrochemicals diversity would still be higher.

Our results show that the diversity patterns of plant species in and around wetlands are the result of both the heterogeneity of these wetlands at the landscape scale and human activities related with their management. Although wetlands are isolated systems, their heterogeneity and diversity have been attributed largely to the interaction with adjacent terrestrial and other aquatic ecosystems such as flooded and riparian forests (Mitsch & Gosselink, 2015; Pott & Vila da Silva, 2015), elements that contribute to connect and maintain diversity in human-dominated landscapes as the Meta Piedmont (Molano, 1998; Rangel, 1998; Minorta-Cely & Rangel-Ch, 2014).

The results of this study support the importance of small forest fragments to conservation in human-dominated landscapes (e.g., Flinn, Lechowicz & Waterway, 2008; Arroyo-Rodríguez et al., 2009). Despite the small size of the fragments of vegetation (<11 ha on average), the diversity of plants found is astonishing. In highly modified landscapes, conservation and management of a large number of small fragments can increase the differentiation in composition and reduce the probabilities of extinction. Similar results were found by Arroyo-Rodríguez et al. (2009) in Los Tuxtlas (Mexico), where even fragments less than 5 ha were of great value to the maintenance of regional diversity. Small patches can be used as stepping stones that increase the landscape’s connectivity and facilitate movements among patches, and they may have complementary or supplementary resources that may favor survival of many animal species in fragmented landscapes (e.g., Brotons, Mönkkönen & Martin, 2003; Pasinelli et al., 2008). Other authors have already highlighted the importance of small wetlands to regional plant diversity (Richardson et al., 2015), not only of natural wetlands (Flinn, Lechowicz & Waterway, 2008) but also of small created wetlands (Chester & Robson, 2013). Therefore, maintaining a diverse set of created wetlands can mitigate the reduction of diversity associated to natural wetlands loss, serving as shelter for birds and other organisms (e.g., Canals et al., 2011; Hapner et al., 2011). Similarly, they can contribute to hydrological dynamics and serve as areas for future ecological restoration (Moreno-Mateos et al., 2015).

### Species composition and future scenarios

In our study compositional similarity was low (<0.5) compared to other studies in small forest patches in human modified landscapes, where higher CS-values were related to homogenization, and lower values to differentiation processes (see details in Arroyo-Rodríguez et al., 2013). The low compositional similarity values found can be explained by differences in type and origin of wetlands, being interesting that compositional similarity increased in most cases by comparing the dominant species (i.e., of the most abundant species), which makes clear the role of management of wetlands as a factor that determines taxonomic differentiation. We found higher differences in species composition within the wetland categories than among them, for both, origin and management. This pattern can be attributed to the high heterogeneity of wetland conditions, maybe due to external ecological factors and different disturbance levels (Clément & Proctor, 2009). Other wetland diversity studies also reported high beta diversity related to spatial heterogeneity (Flinn, Lechowicz & Waterway, 2008; Neiff et al., 2011). This could be attributed to the fact that in wetland remnants the habitat heterogeneity is the main driver for species richness, besides other attributes as its size (Shi et al., 2010). The naturally patchy distribution of wetlands also could contribute to a lower compositional similarity in these ecosystems (Dornelas, Soykan & Ugland, 2011).

An important component in the composition of species in these lentic wetlands was that 19% corresponded to introduced plant species, half of them are exotic species from outside of Colombia, and 13 have invasive potential (Cárdenas, Castaño & Cárdenas, 2010). Species introduction in this region is the result of: (1) planting of ornamental species as shade trees, flower beds and fruit trees for the embellishment predominantly of artificial and mixed wetlands (SNL, CL and FF); (2) sowing of forage species for livestock and, (3) the introduction of aquatic plants to decorate the lakes (CL) and for aquiculture uses (FF). Therefore, management of wetlands determines the change of diversity with the introduction of exotic and native species. As it is known, the introduction of alien species is considered a threat to biodiversity (Alvarado & Gutiérrez, 1997; Cárdenas, Castaño & Cárdenas, 2010), and the ecological integrity of wetlands (MMA, 2002; Gallardo et al., 2015) is one of the major drivers of biotic homogenization (Chen et al., 2010). That is why it requires the implementation of protocols for invasive species management in the creation and maintenance of wetlands (e.g., gardens, landscaping, fences living and restoration).

We suggest two opposed scenarios that may explain current plant diversity patterns in these lentic wetlands and may potentially contribute to understanding future conservation scenarios: (1) diversity is high because of the landscape heterogeneity, similar to the natural diversity pattern before fragmentation, and patches are sufficiently connected in order to maintain diversity in the long term; and (2) the low compositional similarity is a sign of independent dynamics of the wetland communities, hence plant populations are not sufficiently connected, increasing the probability that rare species may disappear from most wetlands (Gutzwiller & Flather, 2011). This could decrease both local and regional diversity. Homogenization processes may cause local extinction of some species and lead to the dominance of few others (McKinney & Lockwood, 1999; Arroyo-Rodríguez et al., 2013).

### Management and conservation

Our results suggest that created lentic wetlands (i.e., artificial and mixed) can have a key role in plant diversity conservation by increasing the available habitat in the region for a variety of typical species. Nevertheless, the plant diversity in natural wetlands may not be replaced or equaled by artificial wetlands, yet the human-made wetlands are a way of safeguarding biodiversity and partially compensate against rapid loss and transformation of natural wetlands (Yoon, 2009; Ghermandi et al., 2010). Created wetlands have the capacity to sustain biodiversity or mitigate negative effects of wetland degradation (Galatowitsch & Van der Valk, 1996; Chester & Robson, 2013), but in some cases their high diversity is the product of more early-successional stage species (Heaven, Gross & Gannon, 2003).

Currently the Piedmont is a highly fragmented landscape (Romero-Ruiz et al., 2004; Etter, McAlpine & Possingham, 2008; Romero-Ruiz et al., 2009) and conservation strategies for wetlands and forests have been reduced and inefficient. A new conservation model could be the promotion of ‘archipelago reserves’ (Halffter, 2007). This approach is particularly useful in highly modified landscapes with high beta diversity, which could integrate the variability of the landscape and allow the conservation of high heterogeneity and plant diversity found in the region (Halffter, 2005; Halffter, 2007). Therefore, we strongly recommend including all wetland types into conservation and management programs, considering how each of them is already contributing to landscape diversity, and how some of them could be improved under appropriate management goals. Also, we suggest the implementation of conservation and restoration strategies according to the wetland type as well as the promotion of local policy for their regulation and management, integrating wetland ecology (Mitsch & Gosselink, 2015) and sustainable utilization, particularly through promoting reconciliation between human land use and biodiversity from integration of the different social actors (i.e., citizens, stakeholders, policy makers and scientists). All these proposals are a way of safeguarding plant diversity of the Meta Piedmont forest species and the lentic wetlands.

##  Supplemental Information

10.7717/peerj.2267/supp-1Table S1Sample coverage ($\hat {C}m$), species richness (^0^*D*) and dominant species diversity (^2^*D*), and relative logarithmic inequality of woody (W) and aquatic (A) plants($\hat {C}m$ is expressed as a percentage of completeness, values from 0 represent no completeness and values of 100 maximum completeness based on Chao & Jost (2012). *only one single individual was found in this wetland, and it was excluded from the RLI analysis; -: in this wetland no plants in this formation were found during sample).

10.7717/peerj.2267/supp-2 Table S2 Local alpha diversity of wetlands (*α*_*w*_) corresponding to the sum of richness of species of woody and aquatic plants in each wetland

10.7717/peerj.2267/supp-3Table S3Total (*α*) and mean ($\bar {\alpha }$ ± SD) alpha diversity, and range values of richness (^0^*D*) and dominant species (^2^*D*) of woody and aquatics plants in wetland types and originsClick here for additional data file.

10.7717/peerj.2267/supp-4 Table S4 Compositional similarity (*CS*) of richness (^0^*D*) and dominant species (^2^*D*) of woody and aquatic plants

## Additional Information and Declarations

### Data Availability

The following information was supplied regarding data availability: Table S1. https://figshare.com/s/e265c23a9171c68e1ab2. DOI 10.6084/m9.figshare.3180961. Table S2. https://figshare.com/s/4b9e4e06ed8d108cdd5e. DOI 10.6084/m9.figshare.3180985. Table S3. https://figshare.com/s/7974fc8c3e9e2046da9a. DOI 10.6084/m9.figshare.3181000. Table S4. https://figshare.com/s/513f4b9de2a74e17aaad. DOI 10.6084/m9.figshare.3181003.
