# Supplementary material for: Effect of wetland management: are lentic wetlands refuges of plant-species diversity in the Andean–Orinoco Piedmont of Colombia?
Source: PeerJ. 2016 Aug 16;4:e2267. doi: 10.7717/peerj.2267 (PMC4991869; doi:10.7717/peerj.2267)
Supplement: Table S1 — ($\hat {C}m$C ˆm is expressed as a percentage of completeness, values from 0 represent no completeness and values of 100 maximum completeness based on Chao & Jost (2012). *only one single individual was found in this wetland, and it was excluded from the RLI analysis; -: in this wetland no plants in this formation were found during sample). [file peerj-04-2267-s001.docx]

**Supplemental Information**

**Table S1. Sample coverage (*Ĉm*), species richness (*^0^D*) and dominant species diversity (*^2^D*), and relative logarithmic inequality of woody (W) and aquatic (A) plants.** (*Ĉm* is expressed as a percentage of completeness, values from 0 represent no completeness and values of 100 maximum completeness based on [Chao & Jost (2012)](#_ENREF_17). *only one single individual was found in this wetland, and it was excluded from the RLI analysis; -: in this wetland no plants in this formation were found during sample).

| **Diversity** | **Woody** | | | | **Aquatic** | | | |
| --- | --- | --- | --- | --- | --- | --- | --- | --- |
| **I. Gamma diversity** | ***Ĉm*** | **^0^*D*** | **^2^*D*** | **RLI_0,2_** | ***Ĉm*** | **^0^*D*** | **^2^*D*** | **RLI_0,2_** |
| 1. All wetlands (γ) | 99 | 407 | 53 | 0.34 | 99 | 99 | 31 | 0.25 |
| 2. Wetland type (α_t_) |  |  |  |  |  |  |  |  |
| Swamps (SW) | 98 | 168 | 42 | 0.27 | 92 | 64 | 33 | 0.16 |
| Heronries (HC) | 98 | 78 | 8 | 0.52 | 96 | 24 | 12 | 0.22 |
| Rice fields (RF) | 95 | 68 | 15 | 0.35 | 96 | 31 | 22 | 0.10 |
| Semi-natural lakes (SNL) | 97 | 201 | 49 | 0.27 | 93 | 53 | 20 | 0.25 |
| Constructed lakes (CL) | 97 | 116 | 17 | 0.40 | 94 | 35 | 19 | 0.17 |
| Fish farms (FF) | 97 | 212 | 37 | 0.32 | 97 | 50 | 23 | 0.20 |
| 3. Wetland origin (α_t_) |  |  |  |  |  |  |  |  |
| Natural | 99 | 195 | 28 | 0.37 | 96 | 70 | 28 | 0.21 |
| Mixed | 97 | 234 | 57 | 0.26 | 96 | 66 | 24 | 0.24 |
| Artificial | 98 | 255 | 37 | 0.35 | 98 | 64 | 27 | 0.20 |
| **II. Alpha diversity** |  |  |  |  |  |  |  |  |
| 4. Each wetland (α_w_) |  |  |  |  |  |  |  |  |
| SW1 | 95 | 19 | 8 | 0.29 | 79 | 18 | 12 | 0.13 |
| SW2 | 93 | 27 | 6 | 0.46 | 98 | 7 | 5 | 0.15 |
| SW3 | 98 | 48 | 19 | 0.24 | 82 | 20 | 13 | 0.14 |
| SW4 | 95 | 48 | 19 | 0.24 | 74 | 23 | 15 | 0.14 |
| SW5 | 96 | 71 | 31 | 0.19 | 98 | 10 | 9 | 0.06 |
| SW6 | 100 | 48 | 18 | 0.25 | 85 | 17 | 11 | 0.17 |
| HC1 | 87 | 14 | 5 | 0.39 | 93 | 10 | 6 | 0.26 |
| HC2 | 97 | 36 | 4 | 0.61 | 94 | 16 | 12 | 0.12 |
| HC3 | 99 | 14 | 1 | 1.00 | 95 | 6 | 4 | 0.21 |
| HC4 | 97 | 35 | 9 | 0.38 | - | - | - | - |
| RF1 | 95 | 28 | 7 | 0.42 | 81 | 20 | 13 | 0.14 |
| RF2 | 89 | 39 | 24 | 0.13 | 100 | 18 | 14 | 0.09 |
| RF3 | 82 | 32 | 10 | 0.34 | 100 | 13 | 9 | 0.13 |
| SNL1 | 79 | 81 | 39 | 0.17 | 94 | 9 | 6 | 0.19 |
| SNL2 | 92 | 39 | 6 | 0.51 | 95 | 11 | 7 | 0.18 |
| SNL3 | 90 | 31 | 10 | 0.33 | 83 | 15 | 10 | 0.14 |
| SNL4 | 95 | 68 | 18 | 0.31 | 83 | 15 | 8 | 0.22 |
| SNL5 | 100 | 34 | 14 | 0.25 | 89 | 13 | 8 | 0.21 |
| SNL6 | 96 | 80 | 22 | 0.29 | 94 | 14 | 10 | 0.13 |
| CL1 | 93 | 20 | 3 | 0.63 | 89 | 9 | 5 | 0.27 |
| CL2 | 91 | 30 | 8 | 0.39 | 80 | 8 | 5 | 0.19 |
| CL3 | 91 | 20 | 3 | 0.63 | - | - | - | - |
| CL4 | 97 | 14 | 4 | 0.47 | - | - | - | - |
| CL5 | 97 | 28 | 5 | 0.52 | - | - | - | - |
| CL6 | 100 | 1* | 1* | * | 94 | 10 | 7 | 0.19 |
| CL7 | 100 | 3 | 2 | 0.37 | 95 | 13 | 9 | 0.13 |
| CL8 | 91 | 28 | 6 | 0.46 | 97 | 6 | 5 | 0.16 |
| CL9 | 96 | 34 | 11 | 0.32 | - | - | - | - |
| FF1 | 95 | 66 | 8 | 0.50 | 76 | 20 | 13 | 0.14 |
| FF2 | 95 | 28 | 13 | 0.23 | 93 | 10 | 6 | 0.24 |
| FF3 | 87 | 67 | 12 | 0.41 | 93 | 7 | 4 | 0.29 |
| FF4 | 89 | 58 | 24 | 0.22 | 100 | 6 | 6 | 0.03 |
| FF5 | 83 | 43 | 14 | 0.30 | 87 | 21 | 13 | 0.16 |
| FF6 | 96 | 41 | 17 | 0.24 | 95 | 10 | 6 | 0.22 |
| FF7 | 90 | 42 | 8 | 0.44 | 90 | 14 | 8 | 0.21 |
| FF8 | 96 | 40 | 7 | 0.47 | 88 | 12 | 8 | 0.16 |
| FF9 | 93 | 39 | 13 | 0.30 | 100 | 6 | 5 | 0.12 |
