# Supplementary material for: Effect of wetland management: are lentic wetlands refuges of plant-species diversity in the Andean–Orinoco Piedmont of Colombia?
Source: PeerJ. 2016 Aug 16;4:e2267. doi: 10.7717/peerj.2267 (PMC4991869; doi:10.7717/peerj.2267)
Supplement: Table S2 [file peerj-04-2267-s002.docx]

**Supplemental Information**

**Table S2. Local alpha diversity of wetlands (*α_w_*) corresponding to the sum of richness of species of woody (W) and aquatic (Aq) plants in each wetland (Species: Ty, typical of Piedmont; IE, introduced exotic; NE, native exotic).**

| **Code** | **Name wetland** | **Origin** | **Type** | ***α_w_*** | **W_Ty** | **W_IE** | **W_NE** | **Aq_Ty** | **Aq_IE** | **Aq_NE** |
| --- | --- | --- | --- | --- | --- | --- | --- | --- | --- | --- |
| SW1 | Humedal Aguas Claras | Natural | Swamp | 37 | 18 |  | 1 | 15 | 1 | 2 |
| SW2 | Humedal Catatumbo | Natural | Swamp | 34 | 22 | 4 | 1 | 4 | 2 | 1 |
| SW3 | Humedal Coroncoro | Natural | Swamp | 68 | 46 |  | 2 | 18 | 2 |  |
| SW4 | Humedal Kirpas-Cuerera | Natural | Swamp | 71 | 42 |  | 6 | 18 | 3 | 2 |
| SW5 | Humedal Zuria | Natural | Swamp | 81 | 66 | 1 | 4 | 3 | 7 |  |
| SW6 | Pantano Hotel Marsella | Natural | Swamp | 65 | 46 |  | 2 | 11 | 6 |  |
| HC1 | Garcero La Silvia | Natural | Heronry | 24 | 13 |  | 1 | 8 | 1 | 1 |
| HC2 | Garcero Las Mercedes | Natural | Heronry | 52 | 35 |  | 1 | 11 | 3 | 2 |
| HC3 | Garcero Matayuca | Natural | Heronry | 20 | 13 |  | 1 | 4 | 2 |  |
| HC4 | Garcero Santa Ana | Natural | Heronry | 35 | 33 | 1 | 1 |  |  |  |
| SNL1 | Lago Palicare | Mixed | Semi-natural lake | 90 | 74 | 5 | 2 | 3 | 6 |  |
| SNL2 | Lago Barquitos | Mixed | Semi-natural lake | 50 | 38 |  | 1 | 7 | 2 | 2 |
| SNL3 | Katan | Mixed | Semi-natural lake | 46 | 27 | 3 | 1 | 7 | 7 | 1 |
| SNL4 | Parque Merecure | Mixed | Semi-natural lake | 83 | 57 | 8 | 3 | 11 | 4 |  |
| SNL5 | Represa la libertad | Mixed | Semi-natural lake | 47 | 33 |  | 1 | 10 | 3 |  |
| SNL6 | Lago Bioparque Ocarros | Mixed | Semi-natural lake | 94 | 64 | 8 | 8 | 12 | 1 | 1 |
| RF1 | Hacienda arrocera Carimata | Mixed | Rice field | 48 | 20 | 1 | 7 | 14 | 3 | 3 |
| RF2 | Hacienda arrocera La Silvia | Mixed | Rice field | 57 | 36 |  | 3 | 11 | 5 | 2 |
| RF3 | Hacienda arrocera Providencia | Mixed | Rice field | 45 | 27 |  | 5 | 10 | 3 |  |
| CL1 | Lago condominio Balmoral | Artificial | Constructed lake | 29 | 15 | 5 |  | 7 | 2 |  |
| CL2 | Lago condominio Barú | Artificial | Constructed lake | 38 | 29 | 1 |  | 4 | 2 | 2 |
| CL3 | Finca El Lago | Artificial | Constructed lake | 20 | 17 | 1 | 2 |  |  |  |
| CL4 | Lago Hotel hacienda San José | Artificial | Constructed lake | 14 | 4 | 9 | 1 |  |  |  |
| CL5 | Lago condominio Horizonte | Artificial | Constructed lake | 28 | 20 | 5 | 3 |  |  |  |
| CL6 | La Tonga | Artificial | Constructed lake | 11 | 1 |  |  | 9 |  | 1 |
| CL7 | Lago Santa Lucia | Artificial | Constructed lake | 16 | 2 | 1 |  | 10 | 3 |  |
| CL8 | Lago Hotel Laguna viva | Artificial | Constructed lake | 34 | 22 | 4 | 2 | 1 | 4 | 1 |
| CL9 | Lago Hotel Los Gavanes | Artificial | Constructed lake | 34 | 25 | 6 | 3 |  |  |  |
| FF1 | Piscícola Acuallanos | Artificial | Fish farm | 86 | 64 |  | 2 | 16 | 3 | 1 |
| FF2 | Piscícola Agualinda | Artificial | Fish farm | 38 | 26 |  | 2 | 8 | 2 |  |
| FF3 | Piscícola Esperanza | Artificial | Fish farm | 74 | 65 |  | 2 | 4 | 2 | 1 |
| FF4 | Piscícola Langostinos | Artificial | Fish farm | 64 | 48 | 5 | 5 | 2 | 3 | 1 |
| FF5 | Piscícola Las Brisas | Artificial | Fish farm | 64 | 40 | 2 | 1 | 16 | 4 | 1 |
| FF6 | Piscícola Manantial | Artificial | Fish farm | 51 | 34 | 5 | 2 | 8 | 2 |  |
| FF7 | Piscícola Margaritas | Artificial | Fish farm | 56 | 40 |  | 2 | 12 | 1 | 1 |
| FF8 | Piscícola Sanmarcanda | Artificial | Fish farm | 52 | 31 | 6 | 3 | 7 | 4 | 1 |
| FF9 | Piscícola Unillanos | Artificial | Fish farm | 45 | 36 | 2 | 1 | 2 | 3 | 1 |
