# Supplementary material for: Effect of wetland management: are lentic wetlands refuges of plant-species diversity in the Andean–Orinoco Piedmont of Colombia?
Source: PeerJ. 2016 Aug 16;4:e2267. doi: 10.7717/peerj.2267 (PMC4991869; doi:10.7717/peerj.2267)
Supplement: Table S3 [file peerj-04-2267-s003.docx]

**Supplemental Information**

**Table S3. Total (α) and mean (ᾱ ± SD) alpha diversity, and range values of richness (*^0^D*) and dominant species (*^2^D*) of woody and aquatics plants in wetland types and origins.**

| **Wetland** | **^0^*D* Woody** | | | | **^2^*D* Woody** | | **^0^*D* Aquatic** | | | **^2^*D* Aquatic** | |
| --- | --- | --- | --- | --- | --- | --- | --- | --- | --- | --- | --- |
| **types** | **n** | **α (ᾱ ± SD)** | **Range** | **α (ᾱ ± SD)** | | **Range** | **n** | **α (ᾱ ± SD)** | **Range** | **α (ᾱ ± SD)** | **Range** |
| Swamps (SW) | 6 | 168 (43.5±18.4) | 19-71 | 42 (16.8±9) | | 6-31 | 6 | 64 (15.8±6.1) | 7-23 | 32 (6.1±3.5) | 5.2-15 |
| Heronries (HC) | 4 | 78 (24.8±12.4) | 14-36 | 8 (4.7±3.3) | | 1-9 | 3 | 24 (10.7±5) | 6-16 | 12 (7±3.9) | 4.1-11.5 |
| Rice fields (RF) | 3 | 68 (33±5.6) | 28-39 | 15 (13.7±9.1) | | 7-24 | 3 | 31 (17±3.6) | 13-20 | 22 (12.1±2.4) | 9.3-13.8 |
| Semi-natural lakes (SNL) | 6 | 201 (55.5±23.4) | 31-81 | 49 (18.2±11.7) | | 6-39 | 6 | 53 (12.8±2.4) | 9-15 | 20 (8.2±1.7) | 5.9-10.3 |
| Constructed lakes (CL) | 9 | 116 (19.8±11.8) | 1-34 | 17 (4.8±3.2) | | 1-11 | 5 | 35 (9.2±2.6) | 6-13 | 19 (6.1±1.9) | 4.5-9.2 |
| Fish farms (FF) | 9 | 212 (47.1±13.4) | 28-67 | 37 (12.9±5.3) | | 7-24 | 9 | 50 (11.8±5.6) | 6-21 | 23 (7.7±3.4) | 4-13.3 |
| **Origin** |  |  |  |  | |  |  |  |  |  |  |
| Artificial | 18 | 195 (33.4±18.6) | 1-67 | 28 (8.8±5.9) | | 1-24 | 14 | 70 (10.9±4.8) | 6-21 | 28 (7.1±2.9) | 4-13.3 |
| Mixed | 9 | 234 (48±21.8) | 28-81 | 57 (16.7±10.5) | | 6-39 | 9 | 66 (14.2±3.3) | 9-20 | 24 (9.5±2.6) | 5.9-13.8 |
| Natural | 10 | 255 (36±18.3) | 14-71 | 37 (12±9.4) | | 1-31 | 9 | 64 (14.1±6) | 6-23 | 27 (9.6±3.9) | 4.1-15 |
