# Supplementary material for: Effect of wetland management: are lentic wetlands refuges of plant-species diversity in the Andean–Orinoco Piedmont of Colombia?
Source: PeerJ. 2016 Aug 16;4:e2267. doi: 10.7717/peerj.2267 (PMC4991869; doi:10.7717/peerj.2267)
Supplement: Table S4 [file peerj-04-2267-s004.docx]

**Supplemental Information**

**Table S4. Compositional similarity (*CS*) of richness (*^0^D*) and dominant species (*^2^D*) of woody and aquatic plants.**

| **Compositional similarity (^q^*CS*)** | **Woody** | | **Aquatic** | |
| --- | --- | --- | --- | --- |
| 1. Within each wetland type | **^0^*D*** | **^2^*D*** | **^0^*D*** | **^2^*D*** |
| Swamps – SW | 0.11 | 0.18 | 0.10 | 0.12 |
| Heronries – HC | 0.09 | 0.01 | 0.17 | 0.26 |
| Rice fields - RF | 0.23 | 0.38 | 0.32 | 0.17 |
| Semi-natural lakes – SNL | 0.13 | 0.19 | 0.09 | 0.25 |
| Constructed Lakes – CL | 0.07 | 0.13 | 0.08 | 0.14 |
| Fish farms – FF | 0.12 | 0.19 | 0.14 | 0.21 |
| 2. Among wetland types |  |  |  |  |
| SW-HC-RF-SNL-CL-FF | 0.21 | 0.21 | 0.32 | 0.32 |
| 3. Among all wetlands |  |  |  |  |
| All wetlands | 0.07 | 0.09 | 0.11 | 0.13 |
| 4. Within each wetland origin |  |  |  |  |
| Natural | 0.16 | 0.27 | 0.09 | 0.15 |
| Mixed | 0.11 | 0.14 | 0.12 | 0.16 |
| Artificial | 0.08 | 0.10 | 0.11 | 0.14 |
| 5. Among wetland origins |  |  |  |  |
| Natural-mixed-artificial | 0.34 | 0.50 | 0.51 | 0.50 |
